# Bioinspired Asymmetric Polypyrrole Membranes with Enhanced Photothermal Conversion for Highly Efficient Solar Evaporation

**DOI:** 10.1002/advs.202306833

**Published:** 2023-12-03

**Authors:** Can Gao, Yimeng Li, Lizhen Lan, Qing Wang, Buguang Zhou, Yue Chen, Jiecong Li, Jiansheng Guo, Jifu Mao

**Affiliations:** ^1^ Key Laboratory of Textile Science and Technology Ministry of Education Donghua University Shanghai 201620 China; ^2^ Key Laboratory of Textile Industry for Biomedical Textile Materials and Technology Donghua University Shanghai 201620 China; ^3^ Shanghai Frontiers Science Center of Advanced Textiles Donghua University Shanghai 201620 China

**Keywords:** asymmetric polypyrrole membranes, bioinspired evaporator, omnidirectional photothermal conversion, reduced evaporation enthalpy, solar desalination

## Abstract

Solar‐driven interfacial evaporation (SDIE) has attracted great attention by offering a zero‐carbon‐emission solution for clean water production. The manipulation of the surface structure of the evaporator markedly promotes the enhancement of light capture and the improvement of evaporation performance. Herein, inspired by seedless lotus pod, a flexible pristine polypyrrole (PPy) membrane with macro/micro‐bubble and nanotube asymmetric structure is fabricated through template‐assisted interfacial polymerization. The macro‐ and micro‐hierarchical structure of the open bubbles enable multiple reflections inner and among the bubble cavities for enhanced light trapping and omnidirectional photothermal conversion. In addition, the multilevel structure (macro/micro/nano) of the asymmetric PPy (PPy‐A) membrane induces water evaporation in the form of clusters, leading to a reduction of water evaporation enthalpy. The PPy‐A membranes achieve a full‐spectrum light absorption of 96.3% and high evaporation rate of 2.03 kg m^−2^ h^−1^ under 1 sun. Long‐term stable desalination is also verified with PPy‐A membranes by applying one‐way water channel. This study demonstrates the feasibility of pristine PPy membranes in SDIE applications, providing guidelines for modulation of the evaporator topologies toward high‐efficient solar evaporation.

## Graphical abstract

A seedless lotus‐pod‐inspired asymmetric pristine PPy (PPy‐A) membrane is fabricated via template‐assisted interfacial polymerization for solar‐driven interfacial evaporation. The hierarchical macro/micro open bubbles enhance light absorption of PPy‐A membrane, facilitating omnidirectional photothermal conversion. The unique multilevel structure of PPy‐A membrane induces water evaporation in cluster form, leading to low evaporation enthalpy, achieving highly efficient solar evaporation.

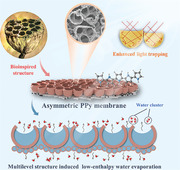

## 1 Introduction

Clean water is considered one of the fundamental and indispensable resources for human survival and development. However, freshwater scarcity is an ongoing challenge faced by communities worldwide.^[^ ^1^ ^]^ It is estimated that by 2025, ≈1.8 billion people will experience water shortages,^[^ ^2^ ^]^ and with the growing global population, the demand for freshwater is expected to increase further. Obtaining fresh water from abundant seawater is regarded as a reliable approach for addressing water demand.^[^ ^3^ ^]^ Desalination technologies, such as membrane distillation,^[^ ^4^, ^5^ ^]^ multi‐stage flash^[^ ^6^ ^]^ and reverse osmosis,^[^ ^7^ ^]^ have been commercialized successfully. Nevertheless, they are not applicable in off‐grid and underdeveloped areas owing to intensive energy requirements and high operating and maintenance costs.^[^ ^8^ ^]^ Solar‐driven interfacial evaporation (SDIE), as an advancing technology, provides cost‐effective and eco‐friendly access to clean water from seawater and wastewater.^[^ ^9^, ^10^, ^11^ ^]^ In comparison to commercialized technologies, SDIE presents notable advantages, such as low cost, free of extra energy input, and flexible installation.

The relentless pursuit of maximizing solar energy utilization is the driving force to improve the performance of SDIE. Efficient solar evaporation is achieved through the excellent thermal localization of photothermal materials at the water–air interface.^[^ ^12^ ^]^ Over the past few years, more than 40 kinds of photothermal materials have been explored for SDIE applications.^[^ ^13^ ^]^ These materials can be generally classified into the following categories: plasmonic,^[^ ^14^, ^15^ ^]^ semiconductor,^[^ ^13^, ^16^ ^]^ polymeric,^[^ ^17^, ^18^ ^]^ and carbonaceous materials.^[^ ^19^, ^20^ ^]^ Notably, carbonaceous materials such as carbon black,^[^ ^21^ ^]^ carbon nanotubes,^[^ ^22^ ^]^ graphene,^[^ ^23^ ^]^ and polymeric materials such as polypyrrole,^[^ ^17^ ^]^ polydopamine,^[^ ^18^ ^]^ and polyaniline^[^ ^24^ ^]^ have been extensively studied due to their broadband light absorption and tunable topological structures.^[^ ^25^ ^]^

Polypyrrole (PPy), a well‐studied conductive polymer, has found extensive application in SDIE due to its photothermal properties. Recent advancements have witnessed numerous investigations into PPy‐based solar evaporators, including deposition of PPy onto various substrates such as paper,^[^ ^26^ ^]^ wood,^[^ ^27^ ^]^ and textiles,^[^ ^28^ ^]^ as well as doping PPy to polymeric networks^[^ ^29^ ^]^ or carbonaceous materials.^[^ ^30^ ^]^ However, considering its unsatisfactory mechanical properties,^[^ ^31^ ^]^ PPy is scarcely used in its pristine form; instead, it is often blended with other materials to form composites for SDIE applications, which may lead to the loss of performance. Considerable studies have demonstrated that surface topology can influence the light absorption and evaporation performance of solar evaporators.^[^ ^15^, ^26^, ^32^ ^]^ Through the design of surface microstructures and the manipulation of their size and shape, it is possible to achieve multiple reflections of light within the microstructures, thereby enhancing light absorption.^[^ ^33^, ^34^ ^]^ Furthermore, the hierarchical nanostructures of hydrogels^[^ ^35^ ^]^ and the meniscuses within 3D structures^[^ ^36^ ^]^ have been demonstrated to promote the activation of water molecules and regulate the enthalpy of evaporation. Previous reports indicate that PPy exhibits adjustable topologies,^[^ ^31^ ^]^ with various structural forms such as nanoparticles,^[^ ^37^ ^]^ nanotubes,^[^ ^38^ ^]^ nanowires,^[^ ^39^ ^]^ and nanosheets^[^ ^40^ ^]^ having been successfully synthesized through diverse methods. In light of this, it is attractive to explore the direct application of pristine PPy in SDIE and investigate the relationship between surface topologies of the PPy membranes and solar evaporation performance.

**Figure 1:**
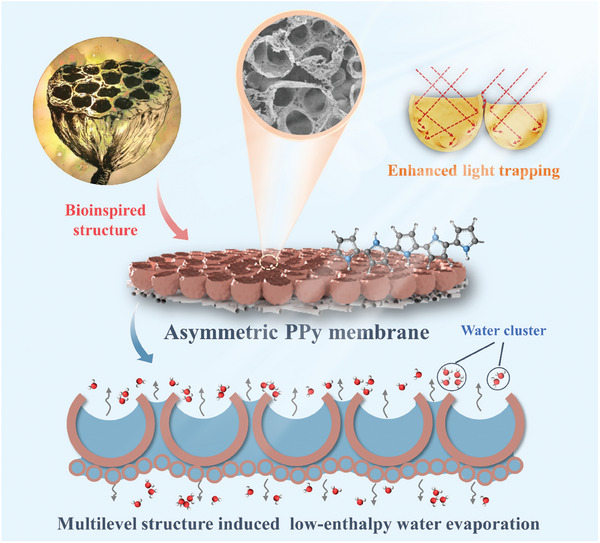
Schematic illustration of seedless lotus‐pod‐inspired PPy‐A membrane with multilevel structure for enhanced light trapping and low‐enthalpy water evaporation, achieving an efficient solar evaporation.

Herein, a flexible pristine asymmetric PPy membrane was prepared via the template‐assisted interfacial polymerization (TIP) method, serving as a free‐standing solar evaporator. The asymmetric PPy (PPy‐A) membranes demonstrated excellent mechanical flexibility and processability, making them directly applicable for SDIE. Inspired by the surface morphology of seedless lotus‐pod, the upper surface of PPy‐A membrane was engineered to feature a unique hierarchical macro/micro‐bubble structure. This innovative structure enabled multiple reflections inner and between the open bubble cavities, markedly enhancing light trapping (**Figure** **1**). The bubble side of PPy‐A membranes showed an impressive light absorbance of 96.3% among the full‐light spectrum and demonstrated robust photothermal conversion at various incident angles of light. The hydrophilicity and substantial capillary channels of PPy‐A membrane facilitated the spontaneous supply of water, promising continuous solar evaporation. The multilevel structure (macro/micro/nano) of PPy‐A membrane induced water evaporation in cluster form, reducing the evaporation enthalpy. Benefiting from these exceptional characteristics, the PPy‐A membranes achieved an impressive water evaporation rate of 2.03 kg m^−2^ h^−1^ under 1 sun, and demonstrated stable performance in seawater desalination. This work provides valuable praxis into the advancement of polymeric membranes for SDIE and underscores the critical role of surface topological engineering in enhancing both photothermal properties and evaporation performance.

## 2 Result and Discussion

### 2.1 Preparation and Characterization of Bioinspired PPy‐A Membranes

**Figure 2:**
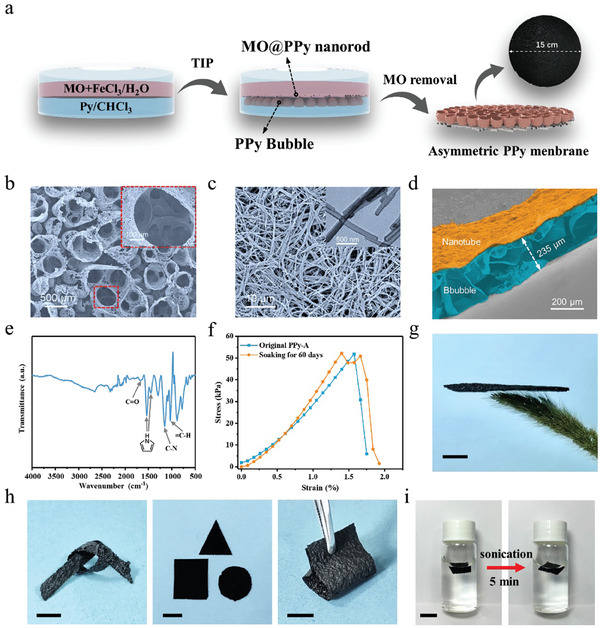
a) Schematics of the synthetic route of asymmetric PPy membrane (PPy‐A) by template‐assisted interfacial polymerization (TIP). SEM images of b) open bubble side; inset is high‐magnification image of single bubble, c) nanotube side; inset is the TEM image of the nanotubes, and d) cross‐section view of PPy‐A membrane. e) FTIR spectra of PPy‐A membrane. f) Stress–strain curves of original PPy‐A membrane and the PPy‐A membrane after soaking in brine for 60 days. g) Digital photograph of the PPy‐A membrane hanging on green bristlegrass (scale bar, 1 cm). h) Digital photograph of PPy‐A with knotted, tailored, and bended configuration (scale bar, 1 cm). i) PPy‐A membrane before and after 5 min sonication (scale bar, 1 cm).

The asymmetric PPy membranes were prepared by modified TIP method according to our previous report.^[^ ^41^ ^]^ The simplified synthesis process is illustrated in **Figure** **2**a. The polymerization of pyrrole at the water/chloroform interface could generate chloroform vapor due to its exothermic behavior,^[^ ^42^ ^]^ inducing the formation of bubble‐like PPy. As the polymerization progressed, the bubble precursor broke or collapsed, allowing pyrrole to continue polymerizing and forming new bubbles,^[^ ^41^ ^]^ resulting in the formation of seedless lotus pod‐like hierarchical bubble structure. The morphology of the bubble side of the PPy‐A membrane observed using scanning electron microscopy (SEM), clearly revealed a multilevel open bubble structure (Figure 2b). A high‐resolution image of the PPy bubble showed the presence of collapsed and broken small bubbles within the larger bubble, further confirming the bubble formation mechanism. On the other side of the PPy‐A membrane, PPy nanotubes, with an approximate diameter of 154 nm, were assembled into arbitrarily oriented bundles with a porous structure (Figure 2c). The mechanism of PPy nanotubes formed in the aqueous phase was explained by the polymerization of pyrrole in self‐degraded template, FeCl_3_/MO complex, which has been carefully studied in previous reports.^[^ ^38^, ^43^ ^]^ The sectional view of the PPy‐A membrane clearly displayed its asymmetric structure, with hierarchical macro/microbubble and nanotube (Figure 2d).

The chemical composition of PPy‐A membrane was analyzed using fourier‐transform infrared (FTIR) spectrometer (Figure 2f). The peaks at 1038 and 1163 cm^−1^ could be attributed to the in‐plane vibration of ═C─H and stretching vibration of C─N. The peaks at 1452 and 1541 cm^−1^ were assigned to the fundamental vibration of the pyrrole ring.^[^ ^44^ ^]^ Additionally, a minor peak at 1680 cm^−1^, associated with stretching mode of C═O, indicated over‐oxidation and propagation process of polymerization reaction involving oxygen and H_2_O.^[^ ^45^ ^]^ The X‐ray photoelectron spectroscopy (XPS) confirmed the presentence of O element in the membrane (Figure S1, Supporting Information). Notably, the PPy‐A membrane demonstrated robust mechanical performance. It displayed a breaking stress of 51.8 kPa and corresponding elongation of 1.58% (Figure 2f). To assess its durability in saline water, the PPy‐A membrane was soaked in 3.5 wt.% of brine for 60 days and subjected to mechanical tests. Impressively, the breaking stress of the PPy‐A membrane remained high at 50.9 kPa, nearly matching the value of the original membrane, with a slight increase in breaking strain to 1.66%. These results demonstrated the strong corrosion resistance of the PPy‐A membrane. What is more, the PPy‐A membrane boasted a lightweight feature, with an area density of 15.1 g m^−2^, making it easily rest upon Setaria viridis fibers (Figure 2g). Large‐sized PPy‐A membrane could be easily achieved through the proposed method according to the specific requirements. Here, we successfully fabricated a PPy‐A membrane with a substantial diameter of 15 cm (Figure 2a), indicating the potential for large‐scale production. The Derjaguin–Muller–Toporov (DMT) modulus of PPy‐A membrane was tested using an atomic force microscopy (AFM), as exhibited in Figure S2 (Supporting Information). The DMT moduli of bubble side and nanotube side of PPy‐A membrane were 1.59 and 1.85 MPa, respectively, indicating its excellent flexibility. Furthermore, the bubble side displayed a smaller modulus, which supports greater deformation. Therefore, the PPy‐A membrane demonstrated remarkable mechanical flexibility and processability, allowing for knotting, cutting, bending, and squeezing, as shown in Figure 2h. More impressively, it was capable of swing in saline water without breaking and damaging even after 60 days immersing in 3.5 wt.% NaCl solution (Video S1, Supporting Information). The robustness of the PPy‐A membrane was evaluated by subjecting it to sonication for a duration of 5 min, during which no observable structural damage or deterioration was detected. The excellent mechanical performance of PPy‐A membrane makes it feasible and reliable for the application in SDIE for solar desalination and wastewater purification.

### 2.2 Light Absorption and Photothermal Performances

**Figure 3:**
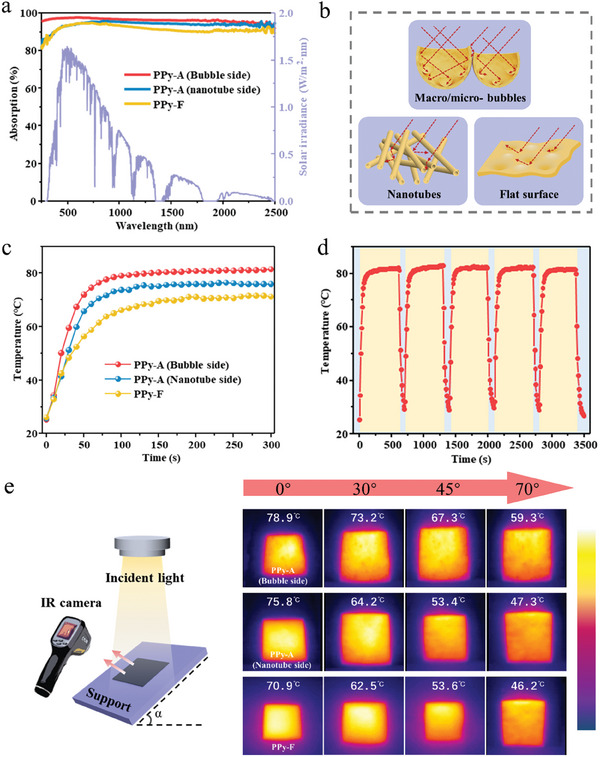
a) Absorption spectra of bubble side and nanotube side of PPy‐A membrane, and PPy‐F membrane. b) Schematic illustration of light trapping of PPy membranes with different surface morphologies. c) Surface temperature change of dry‐state bubble side and nanotube side of PPy‐A and PPy‐F membranes under one sun. d) Cyclic photothermal conversion performance of PPy‐A membrane. e) Schematic of PPy membrane with different tilt angles under one sun irradiation monitored by IR camera, and the recorded IR images of the bubble side and nanotube side of PPy‐A and PPy‐F membranes at different tilt angles.

Efficient light absorption is an essential prerequisite for improving solar energy utilization. Here, we aim to explore how surface morphology affects light trapping and photothermal performance by manipulating the topology structure of PPy membranes. To facilitate comparison, another PPy membrane was prepared (Experimental Section, Supporting Information), with a flat surface morphology on one side (Figure S3a, Supporting Information), which is referred to as PPy‐F. The optical absorption performance of the PPy membranes was evaluated using UV–vis–NIR spectrophotometer equipped with an integrating sphere. The PPy‐F membrane, which lacks specific surface structures, presented a light absorbance of only 92.7% over the full solar spectrum (280–2500 nm), as exhibited in **Figure** **3**a. In contrast, the nanotube side of PPy‐A membrane showed an elevated light absorbance of 94.0%. The enhancement can be attributed to the increased scattering and reflection of light among the pores formed between the nanotubes. Remarkably, the PPy bubbles demonstrated an enhanced light trapping, achieving a high light absorbance of 96.3%. The increase in light absorbance could be primarily attributed to the unique bioinspired architecture of hierarchical PPy bubbles, which facilitated multiple reflections of light within and among the bubble cavities, as illustrated in Figure 3b. This is supported by the measured light reflectance of different PPy surfaces (Figure S4, Supporting Information), where PPy‐A exhibited the lowest reflectance. The disparity in light absorption is directly manifested in the photothermal performance of PPy membranes. As exhibited in Figure 3c, for the same PPy‐A membrane, subjecting its asymmetric sides to light irradiation resulted in notable differences in photothermal performance. The bubble side of the PPy‐A membrane reached a steady‐state temperature of 81.1 °C, while the nanotube side only reached 76.1 °C. In comparison, the flat PPy‐F membrane displayed a lower temperature of 71.1 °C. The cyclic photothermal performance of PPy‐A membrane was further tested by switching on and off the light source multiple times in quick succession. As shown in Figure 3d, the PPy‐A membrane displayed a rapid photothermal response of 120 s and maintained a stable photothermal conversion ability.

In real‐world solar irradiation, the variation in the inclination angle of sunlight can greatly affect the light absorption of the evaporator, leading to fluctuations in the photothermal performance. The steady‐state temperature variations of PPy membranes were examined under different incident angles of illumination. As exhibited in Figure 3e, under vertical irradiation (i.e., α = 0), the PPy membranes reached the steady‐state temperatures corresponding to the values in Figure 3c. As the inclination angle varied from 0° to 70°, the steady‐state temperature of bubble side of the PPy‐A membrane slightly decreased from 78.9 to 59.3 °C, maintaining efficient photothermal conversion ability even with a significant reduction in projected area. This behavior indicates that the hierarchical bubble structure of PPy‐A membrane enabled a wide‐angle light absorption, facilitating efficient photothermal conversion. Conversely, the steady‐state temperature on the nanotube side significantly decreased from 75.8 to 47.3 °C. This drop is attributed to the weakened light trapping within nanotube bundles. In comparison, the PPy‐F membrane exhibited a significant decline in photothermal performance, with steady‐state temperatures dropping from 70.9 to 46.2°C under varying incident angles of light. These findings revealed the significant influence of topological structure on the photothermal performance of the evaporator, highlighting the advantages of the biomimetic‐structured PPy‐A membrane in omnidirectional photothermal conversion.

### 2.3 Solar Evaporation Performance

**Figure 4:**
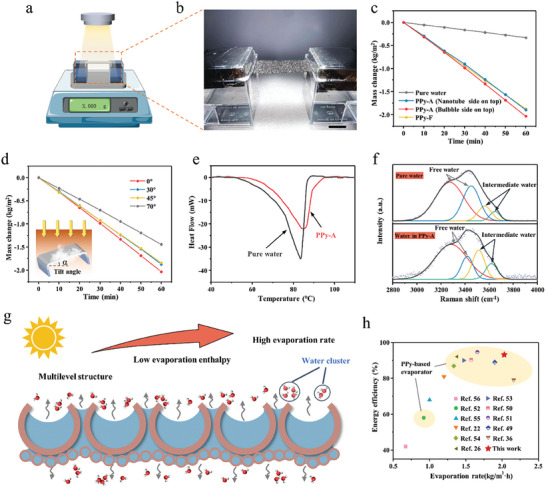
a) Schematic illustration of the solar evaporation setup with PPy‐A membrane hanging between two water boxes. b) Photograph of PPy‐A membrane as the evaporator (scale bar, 1 cm). c) Mass changes of water with PPy‐A (bubble side on top), PPy‐A (nanotube side on top), and PPy‐F under one sun, pure water as control. d) Mass changes of water in PPy‐A at different tilt angles under one sun. e) DSC curves of pure water and wet PPy‐A membrane. f) Raman spectra of pure water and water in PPy‐A membrane fitted with Gaussian function. g) Schematic diagram of evaporation interface of PPy‐A membrane. h) Comparison of evaporation performance of PPy‐A membrane with previous reports.

The solar evaporation performance of PPy‐A membrane was verified through the meticulously designed evaporation device. As illustrated in **Figure** **4**a, the experiment was conducted in a constant indoor environment with a solar simulator as the light source, and the evaporation mass of water was recorded by the balance. The structure of the solar evaporation device with PPy‐A membrane was exhibited in Figure 4b. Thanks to the excellent mechanical flexibility, the PPy‐A membrane was able to be bent and stably suspended between two water boxes, ensuring both sides of the evaporator were completely exposed to the air. This evaporation mode has been proven to improve the evaporation rate through enhanced natural convection.^[^ ^46^ ^]^ Benefiting from the good hydrophilicity of PP‐A membrane (as shown in Figure S5 and Video S2, Supporting Information) and substantial capillary channels formed by macro/micro‐bubbles and nanotubes, water could be spontaneously extracted from the bulk water to the evaporation area by the membrane, ensuring continuous solar evaporation. The evaporation device was employed to compare the evaporation performance of different samples. As shown in Figure 4c, the evaporation rate of pure water in the container was notably low, measuring only 0.33 kg m^−2^ h^−1^. For PPy‐A membrane, we observed varying evaporation rates with different sides of the asymmetric surfaces exposed to illumination. When the nanotube side faced the light source, the PPy‐A membrane achieved an evaporation rate of 1.90 kg m^−2^ h^−1^. In contrast, when the bubbles side was on the top and exposed to light, the evaporation rate of PPy‐A membrane increased to 2.03 kg m^−2^ h^−1^, which is ≈6 times that of pure water and increased by 5.4% compared to the nanotube side. The enhanced evaporation rate can be ascribed to the superior light trapping and photothermal performance resulting from the unique surface morphology of hierarchical PPy bubbles. Inevitably, the PPy‐F, with limited light absorption capabilities, showed a reduced evaporation rate of 1.88 kg m^−2^ h^−1^.

As has been discussed before, the bioinspired multilevel bubbles of PPy‐A membrane exhibited highly efficient photothermal conversion over a wide range of inclination angles of light. The omnidirectional photothermal conversion ability was considered to be beneficial for keeping stable evaporation rate when dealing with significant changes in inclination angles of light. As shown in Figure 4d, when the tilt angle (equivalent to the inclination angle of light) of the evaporation surface varied from 0° to 45°, the evaporation rate of PPy‐A membrane slightly decreased from 2.03 to 1.84 kg m^−2^ h^−1^. Even when the tilt angle further increased to 70°, the evaporation rate remained at 1.44 kg m^−2^ h^−1^ despite a sharp reduction in projected area. According to the research of Liu et al.,^[^ ^47^ ^]^ the effective solar irradiation inclination angle typically ranges from ≈10° to 70° in a day over the course of a day. Therefore, the PPy‐A membrane can ensure efficient solar evaporation under practical irradiation conditions.

To investigate the reasons underlying the efficient evaporation performance of PPy‐A membrane, differential scanning calorimetry (DSC) measurements and evaporation tests were conducted to evaluate the energy consumption of water (i.e., evaporation enthalpy) during liquid–gas phase transition. Notably, both the evaporation and DCS results showed the tendency of reduction in water evaporation enthalpy for the PPy‐A membrane when compared to pure water, as exhibited in Figure 4e and Figure S6 (Supporting Information). Based on the evaporation tests, the evaporation enthalpy of water in PPy‐A membrane was calculated to be 1913.29 kJ kg^−1^ which was much lower than the value of 2441.87 kJ kg^−1^ of pure water, indicating reduced energy consumption for water evaporation in the PPy‐A membrane. Previous reports have confirmed that the hierarchical porous structure of polymeric networks would induce water to evaporate in small clusters composed of several to dozens of molecules, requiring less energy for evaporation.^[^ ^13^, ^35^ ^]^ Raman spectra further revealed the different types of hydrogen bonds in water. As shown in Figure 4f, the free water with four hydrogen bonds was associated with the peaks at 3250 and 3427 cm^−1^, and the weakly hydrogen‐bonded intermediate water corresponded to the peaks at 3514 and 3635 cm^−1^.^[^ ^48^ ^]^ The PPy‐A membrane demonstrated a higher ratio of intermediate water to free water compared to pure water, favoring the cluster evaporation of water.

Moreover, the water aggregation state on the surface of bubble side and nanotube side of PPy‐A membrane and PPy‐F membrane was recorded by microscope during the evaporation (Figure S7, Supporting Information). The microscopic images revealed that on the bubble side of the PPy‐A membrane, water exhibited noticeable stratification, manifesting as irregular water masses segmented by irregular pores and gaps. Similarly, water on the nanotube side of the PPy‐A membrane displayed a comparable state, albeit with smaller water voids. In contrast, the PPy‐F surface was covered with a relatively smooth water layer. Clearly, the aggregation state of water on the evaporator surface is intricately associated with its surface morphology. It can be concluded that the multilevel structured surface of PPy‐A membrane would lead to varied surface tension of water, resulting in the segmentation of water into numerous masses. Consequently, a significant portion of water would evaporate in cluster form, thereby reducing the evaporation enthalpy of water and ultimately improving the evaporation rate under fixed energy input, as illustrated in Figure 4g.

The solar‐to‐vapor energy efficiency (η) could be further calculated by the following equation^[^ ^35^ ^]^:

(1) $$\eta =\frac{\dot{m}h_{v}}{C_{opt}P_{0}}$$

where $\dot{m}$ is the light‐induced water evaporation rate (kg m^−2^ h^−1^) by subtracting natural evaporation rate (Table S2, Supporting Information), *h_v_* is water evaporation enthalpy (kJ kg^−1^), *C_opt_* represents the optical concentration, and *P*_0_ denotes the solar power density of 1 sun. Owing to the limited thermal localization, the pure water reached an efficiency of only 14.5% (Figure S8a, Supporting Information). In comparison, PPy‐F, with efficient thermal localization, achieved an elevated efficiency of 75.9%. Through surface topology modification, the PPy‐A membrane further promoted the solar to vapor energy efficiency, with an efficiency of 86.4% when the nanotube side was under illumination and an even higher efficiency of 93.3% when the bubble side was under irradiation. In summary, the improved solar evaporation performance of the PPy‐A membrane could be primarily attributed to enhanced light trapping, efficient photothermal conversion, and reduced evaporation enthalpy. Benefiting from these advantages, the PPy‐A membrane displayed an outstanding evaporation rate and energy efficiency when compared to previously reported evaporators.^[^ ^22^, ^26^, ^36^, ^49^, ^50^, ^51^, ^52^, ^53^, ^54^, ^55^, ^56^ ^]^

**Figure 5:**
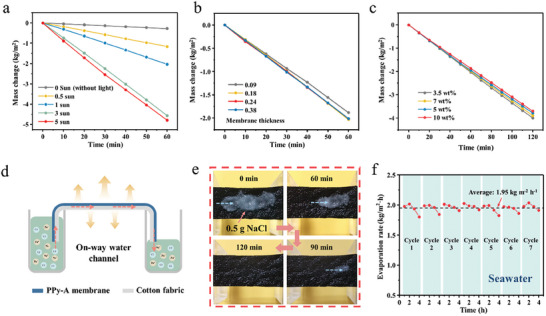
a) Mass change of water with PPy‐A membrane under different light intensities (0, 0.5, 1, 3, and 5 sun). b) Mass change of water of PPy‐A membrane under 1 sun with different membrane thickness. c) Mass change of water with PPy‐A membrane in different brine salinity under 1 sun. d) Schematic of one‐way water channel with PPy‐A membrane for brine evaporation. e) Dissolution of 0.5 g NaCl on the surface of PPy‐A membrane with one‐way water channel in the dark. f) Cyclic evaporation performance of PPy‐A membrane in seawater under 1 sun.

The evaporation performance of PPy‐A membrane under different light intensities was further measured. As shown in **Figure** **5**a, even under 0.5 sun, the PPy‐A membrane achieved an evaporation rate of 1.16 kg m^−2^ h^−1^, which is particularly advantageous for practical solar desalination in case of insufficient solar intensity caused by environmental factors or transmission decline through the condensing cover. When the solar intensity further increased to 3 sun, the evaporation rate of PPy‐A membrane was greatly promoted to 4.56 kg m^−2^ h^−1^. However, with a further increase in light intensity to 5 sun, there was no substantial increase in the evaporation rate, which remained at 4.78 kg m^−2^ h^−1^. As exhibited in Figure S9a (Supporting Information), the energy efficiency of the PPy‐A membrane was 75.6% under 3 sun, but it decreased significantly to only 47.5% under 5 sun. While the higher energy density at 5 sun results in increased thermal losses, the substantial drop in the evaporation performance of the PPy‐A membrane is primarily attributed to inadequate water supply. As displayed in IR images in Figure S10 (Supporting Information), owing the insufficient water supply, the central area of PPy‐A membrane under 5 sun possessed extremely high temperatures. A mismatch between the fast evaporation and insufficient water supply can lead to reduced efficiency. Notably, in typical outdoor conditions, solar radiation generally falls below 1 sun, which implies that our samples exhibit higher energy efficiency in relatively low‐irradiation outdoor environments. In addition, the influence of membrane thickness of PPy‐A on evaporation performance was further investigated. By controlling the polymerization time, we prepared PPy‐A membranes with thicknesses of 0.09, 0.18, 0.24, and 0.38 mm, respectively, as exhibited in Table S3 (Supporting Information). When the polymerization time was 24 h, the membrane thickness was merely 0.09 mm and it exhibited the lowest evaporation rate and energy efficiency (Figure S9b, Supporting Information). This was mainly associated with insufficient water supply, as explained in previous studies.^[^ ^57^ ^]^ As the polymerization time was extended to 36 h, the membrane thickness increased to 0.18 mm, the evaporation rate and efficiency were improved to 2.01 kg m^−2^ h^−1^ and 92.2%, respectively. Further extension of the polymerization time to 50 and 96 h increased the membrane thickness to 0.24 and 0.38 mm, respectively. However, the resulted evaporation performance presented limited enhancement, indicating that the water supply had become well‐matched with the evaporation rate. Considering that a 96 h polymerization time was excessively long and did not yield significant energy efficiency gains, adopting the PPy‐A membrane with a 50 h polymerization time is a more reasonable choice.

What is more, PPy‐A membrane is capable of handling brine with concentrations of up to 10.0 wt.% (Figure 5b). With the brine salinity ranging from 3.5 to 10.0 wt.%, the evaporation rate of PPy‐A membrane showed only a minor reduction from 2.00 to 1.85 kg m^−2^ h^−1^ with a small reduction of 7.5% under 1 sun. The corresponding energy efficiency of the solar evaporation for 10.0 wt.% brine remained high at 83.1% (Figure S9c, Supporting Information), demonstrating the exceptional durability of PPy‐A membrane in brine evaporation. Nevertheless, the issue of salt crystallization still remains a challenge due to the fast water evaporation during solar evaporation and long‐term desalination process.^[^ ^58^, ^59^ ^]^ The accumulated salt will block the water channels and reduce light absorption, resulting in continuous decline in evaporation rate, and even damage and destroy the evaporator. To mitigate the impact of salt accumulation, it is crucial to employ a rational evaporator structure^[^ ^60^ ^]^ and optimize the evaporation mode.^[^ ^61^ ^]^ As shown in Figure 5d, we introduced a modified evaporation mode with one‐way water channel by adjusting water level difference and enhancing capillary water transport with cotton fabric. In such mode, water can be spontaneously promoted to flow from the high‐level side to the low‐level side through the water channel. The generated water potential difference can enhance the directional flow of water and thereby reduce salt accumulation. Water flowing process was observed through salt dissolution by uniformly spreading 0.5 g of salt on the surface of the PPy‐A membrane (Figure 5e). It was observed that the salt gradually dissolved on the PPy membrane from left to right, indicating a higher water flux near the high water level side, where salt particles dissolved quickly. Conversely, near the low water level side, salt dissolution was lower, suggesting the water flowed directionally from high‐level side to the low‐level side. The data demonstrated that the one‐way water channel significantly accelerated salt particle dissolution, with 0.5 g of salt completely dissolving within 2 h. In comparison, in the case of double‐side water supply (Figure S11, Supporting Information), the dissolution time for 0.5 g of salt was prolonged to 8 h. It can be anticipated that with the one‐way water channel mode, the PPy‐A membrane not only reduces salt accumulation but also efficiently self‐cleans the surface from salt particles in the absence of light, which is highly desirable for long‐term evaporation. In 7‐cycle solar evaporation experiments using seawater, the PPy‐A membrane demonstrated an average evaporation rate of 1.95 kg m^−2^ h^−1^, exhibiting its long‐term stability in seawater desalination (Figure 5f).

### 2.4 Outdoor Solar Desalination and Wastewater Purification

**Figure 6:**
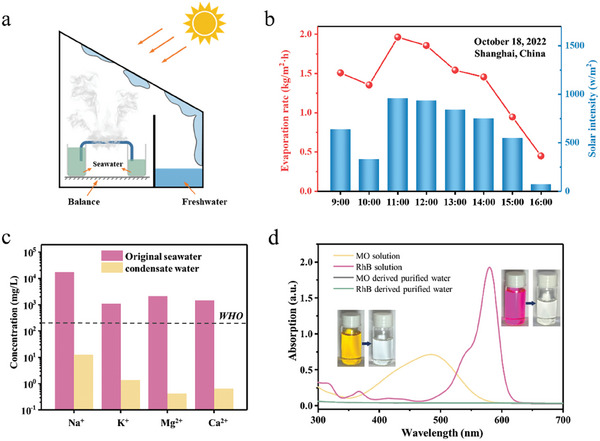
a) Schematic of outdoor seawater (obtained from the Yellow Sea) desalination device. b) Time‐dependent evaporation rate of PPy‐A membrane and solar intensity. c) Primary ion concentrations in seawater before and after desalination. d) UV–vis spectra and digital images (inset) of MO and RhB before and after wastewater purification with PPy‐A membrane.

To assess the actual outdoor solar desalination performance, the PPy‐A membrane was integrated into a sealed one‐slope distiller, as shown in **Figure** **6**a. The vapor generated during the evaporation process condensed on the cover of the solar still and was subsequently collected as it flowed into the freshwater tank. The evaporation rate could be calculated by recording the mass of water at different time intervals using a balance. The outdoor experiments were conducted with real seawater at the campus of Donghua University (31° E, 121° N) in Shanghai, China. The photograph of the desalination device is provided in Figure S12a (Supporting Information). Throughout the desalination process, data on the evaporation rate, solar intensity (Figure 6b), as well as the environment temperature and humidity (Figure S12b, Supporting Information) were recorded. It could be observed that under an average solar irradiance of 634.1 W m^−2^, the PPy‐A membrane achieved an average evaporation rate of 1.38 kg m^−2^ h^−1^, demonstrating its excellent freshwater production capability. Furthermore, under the highest solar irradiance (958 W m^−2^) of the day, the PPy‐A membrane reached an evaporation rate of 1.96 kg m^−2^ h^−1^, closely approaching the rates observed in indoor experiments. Over the course of 8 h solar desalination, the total evaporation mass achieved 11.1 L m^−2^, which is enough to support the daily drinking water needs of a normal family with 1 m^−2^ membrane.

To evaluate the water quality, the concentrations of ions in both original seawater and condensed water were measured with inductively coupled plasma spectroscopy (Prodigy‐ICP). As shown in Figure 6c, four typical ions (Na^+^, K^+^, Mg^2+^, and Ca^2+^) markedly reduced by three to four orders of magnitude after condensation, meeting the WHO standard for drinking water.^[^ ^62^ ^]^ In addition, the SDIE is also commonly applied for wastewater purification in various settings. To assess the wastewater purification capabilities of the PPy‐A membrane, two types of simulated wastewater containing MO and RhB were used as model pollutants. The purified water was collected using the distillation system and compared with original wastewater. The UV–vis spectra showed that the characteristic peaks of RhB and MO both disappeared (Figure 6e), indicating the complete removal of the pollutants in purified water. The excellent solar desalination and wastewater purification performance of PPy‐A membrane verifies its potential in real‐world applications.

In this study, we present a case of customized structured pristine PPy membranes for SDIE. Comparative investigation is conducted to analyze the impact of different surface topologies on photothermal performance. Within the range of surface topologies, we have chosen hierarchical macro/micro‐bubble structure, nanotube structure, and planar structure, to assess their effects on photothermal performance. Based on the results of light absorption, we conclude that the hierarchical macro/micro‐bubble structure facilitates multiple internal reflections of light, thereby enhancing light absorption. Subsequent experiments confirm the superior photothermal performance of the hierarchical macro/micro‐bubble structure and its unexpected ability to reduce the evaporation enthalpy of water. These findings shed light on the connection between evaporator structure and performance, providing valuable insights for the design of future solar evaporators.

## 3 Conclusion

In conclusion, a seedless lotus‐pod‐inspired pristine PPy‐A membrane with an asymmetric structure was prepared through a modified TIP method. The PP‐A membrane exhibited excellent mechanical flexibility, promising its application in SDIE. The biomimetic hierarchical macro/micro‐bubble structure endowed the PP‐A membrane with enhanced optical performance, achieving a light absorbance of 96.3% and omnidirectional photothermal conversion. The unique multilevel structure of the PPy‐A membrane induced water evaporation in the form of clusters which reduced the evaporation enthalpy of water. As a result, the PPy‐A membrane achieved an outstanding evaporation rate of 2.03 kg m^−2^ h^−1^ with an energy efficiency of 93.3%. In addition, the optimized evaporation mode with one‐way water channel greatly reduced salt accumulation on PPy‐A membrane, ensuring long‐term stability in brine evaporation. This work provides novel insights for the synthesis and application of polymeric photothermal materials in SDIE and demonstrates a successful strategy for enhancing evaporation performance through the manipulation of surface topologies.

## Conflict of Interest

The authors declare no conflict of interest.

## Supporting information

Supporting Information

Supplemental Video 1

Supplemental Video 2

## Data Availability

The data that support the findings of this study are available from the corresponding author upon reasonable request.
