# Supplementary material for: Bioinspired Asymmetric Polypyrrole Membranes with Enhanced Photothermal Conversion for Highly Efficient Solar Evaporation
Source: Adv Sci (Weinh). 2023 Dec 3;11(6):2306833. doi: 10.1002/advs.202306833 (PMC10853741; doi:10.1002/advs.202306833)
Supplement: Supplementary file 1 — Supporting Information [file ADVS-11-2306833-s002.pdf]

ADVANCED SCIENCE
Open Access

Supporting Information

for *Adv. Sci.*, DOI 10.1002/advs.202306833

Bioinspired Asymmetric Polypyrrole Membranes with Enhanced Photothermal Conversion for Highly Efficient Solar Evaporation

Can Gao, Yimeng Li, Lizhen Lan, Qing Wang, Buguang Zhou, Yue Chen, Jiecong Li, Jiansheng Guo and Jifu Mao**

Supporting Information

Bioinspired asymmetric polypyrrole membranes with enhanced photothermal conversion for highly efficient solar evaporation

Can Gao [a, 1], *Yimeng Li* [a, b, 1], *Lizhen Lan* [a, b, 1], *Qing Wang* [a], *Buguang Zhou* [a], *Yue Chen* [a], *Jiecong Li* [a], *Jiansheng Guo* [a, *], *Jifu Mao* [a, b, c, *]

[a] Key Laboratory of Textile Science and Technology, Ministry of Education, Donghua University, Shanghai 201620, China

[b] Key Laboratory of Textile Industry for Biomedical Textile Materials and Technology, Donghua University, Shanghai 201620, China.

[c] Shanghai Frontiers Science Center of advanced Textiles, Donghua University, Shanghai 201620, China

*Corresponding authors:

E-mail addresses: Jifu.mao@dhu.edu.cn (Jifu Mao), jsguo@dhu.edu.cn (Jiansheng Guo).

College of Textiles, Donghua University, 2999 North Renmin Road, Songjiang District, Shanghai 201620, China.

Experimental Section

Materials

Pyrrole (Py, 99%) was purchased from Wendong Chemical Co., Ltd. (Shanghai, China). Iron trichloride hexahydrate ($FeCl_3 \cdot 6H_2O$), chloroform, Tween-80 (T80), methyl orange (MO), rhodamine B (RhB), anhydrous ethanol, and sulfuric acid (H_2SO_4) were obtained from Sinopharm Chemical Reagent Co., Ltd. (Shanghai, China). Sodium sulfosalicylate (NaSSA) and NaCl were obtained from Rhawn (Shanghai, China). Seawater was obtained from the Yellow Sea (Weihai, China). All chemicals were used as received.

Fabrication of PPy-A membrane

The PPy-A membranes were synthesized by a template-assisted interfacial polymerization (TIP) method. The fabrication process was followed and modified with our previous study [1]: 18.2 g of $FeCl_3 \cdot 6H_2O$ was added to 320 mL of deionized water containing 5 mM of MO, and the solution was stirring for 30 min. Next, 3 mL of pyrrole monomer was dropped to 150 mL of chloroform. $FeCl_3$/ MO mixed solution was then transferred on to the top of Py/ chloroform solution to initiate the polymerization. After reaction for 50 h at 4°C, the membrane was collected and washed with 1 M of HCl in 70% ethanol solution, and deionized water until the solution became colorless and neutral. The obtained PPy-A membranes were dried at room temperature.

Fabrication of PPy-F membrane

The PPy-F membrane was prepared as our previous report [2]. Specifically, 18.2 g $FeCl_3 \cdot 6H_2O$ and 13.4 g NaSSA were added into 320 mL DI water containing T80 (3.375

g/L) and stirred for 30 min. The mixed solution was then transferred on to the top of 150 mL of chloroform containing Py (0.29 M) for polymerization. After reaction for 40 h at 4°C, the obtained membranes (PPy-F) were collected and washed with 1 M of HCl in 70% ethanol solution and DI water, and dried at room temperature. In all experiments, the flat side of the PPy-F membrane was exposed to light.

Characterization

The morphology of the PPy membranes was observed by a field scanning electron microscope (SEM, Hitachi SU8010, Japan) and transmission electron microscopy (TEM, JEOL model JEM-1230, Tokyo, Japan). Fourier-transform infrared (FTIR) spectrum (Thermofisher Antaris II, USA) and X-ray photoelectron spectrometer (XPS, Thermofisher Escalab250xi, USA) were conducted to analyze the chemical composition of the PPy-A membrane. The tensile properties of the membranes were tested using a Single Fiber Strength Tester (YG-001, Wenzhou Fangyuan Instrument Co., Wenzhou, China) at 30 mm gauge length and 10 mm/min crosshead speed. The DMT modulus was tested with an atomic force microscopy (Dimension FastScan, Bruker, German). The light absorption of the PPy membranes was measured by UV-vis-NIR spectrophotometer (UV3600, Shimadzu, Japan) with an integrating sphere accessory. The light absorbance was calculated by A=1-R-T, where R and T were the reflectance and transmittance of samples respectively. Raman spectra were obtained by a Raman Imaging Microscope (DXR2xi, USA) with 532-nm laser. The UV-vis absorption spectra were obtained using a UV-vis spectrophotometer (Lambda 35, PerkinElmer, USA). The solar irradiation was provided by a solar simulator (CME-

Xe300F, Beijing, China). The ion concentrations were measured with an inductively coupled plasma spectroscopy (Prodigy-ICP). The temperature distribution was monitored by an infrared camera (FLIR-TG165, 0.1°C) and thermocouples. Differential scanning calorimetric (DSC) measurements were performed on a differential scanning calorimetry (DSC8500, PerkinElmer).

Photothermal conversion of PPy membranes with various inclination angles

The PPy membranes were located on an insulation foam as a support. The simulated sunlight illuminated with fixed vertically direction. The inclination angle of light on the membranes was controlled by changing the tilt angle of the support. The steady-state temperature of the PPy membranes at different tilt angles was recorded by the IR camera.

Solar-driven steam generation of PPy membranes

Solar evaporation measurements were conducted in a constant indoor environment with a temperature of 25°C and humidity of 50%. The PPy membranes were tailored into a standard size of 2 cm × 10 cm. As shown in Figure 4a, two ends of membranes were immersed in water boxes and the evaporation part of membranes was hanging between the water boxes, allowing for double side evaporation. The evaporation device was placed on the balance for real-time monitoring of water evaporation. The mass change of water was recorded by the balance every 10 min. The irradiation was provided by the solar simulator.

Solar desalination and wastewater purification based on the PPy-A membrane

The solar desalination experiments were conducted with a homemade distiller. A large

PPy-A membrane with a size of 6 cm × 8 cm of the evaporation area was used for the outdoor solar desalination. The real seawater was fed into the water boxes for desalination. The quality of desalinated water was evaluated by detecting the ions concentration of Na^+, K^+, Mg^{2+}, Ca^{2+} in desalinated water compared with the original seawater.

Statistical Analysis

Statistical analysis was performed using GraphPad Prism Software 8.0. Data are presented as the mean ± SD. One-way analysis of variance (ANOVA) where was used to statistically analyze the data. Statistical significance was set at $p < 0.05$. The ns in figures represents nonsignificant.

To evaluate the purification capability of PPy-A membrane, two model pollutants: MO and RhB were used in simulated wastewater. The wastewater containing 10 mg ml^{-1} of MO or RhB was fed into the water boxes for evaporation and then the vapor was condensed and collected to obtain the purified water. The purification performance was assessed by measuring the characteristic peaks of the pollutants using a UV-vis spectrophotometer.

Estimation of water evaporation enthalpy

The water evaporation enthalpy h_v could be calculated by a classical semi-empirical equation [3]:

$$h_v = \mathrm{a} + \mathrm{bT} + \mathrm{CT}^{1.5} + dT^{2.5} + eT^3 \tag{S1}$$

where a = 2500.304, b = -2.2521025, c = -0.021465847, d = 3.1750136×10^4, and e =

-2.861759×10^5. Based on Equation (S1), the evaporation enthalpy of pure water and water in PPy-A membrane were calculated to be 2430.97 and 2410.49 kJ kg^{-1} respectively. The corresponding energy efficiencies for pure water and PPy-A membrane were 14.5% and 118% respectively under 1 sun. The calculated energy efficiency of PPy-A membrane exceeded 100%, which is in inconformity with the law of energy conservation. The results indicate that the evaporation mode of water in PPy-A membrane may not be accurately described by traditional model owing to the alteration in water state during evaporation. Therefore, we came to investigate the evaporation enthalpy of pure water and water in PPy-A membrane by two methods: DSC measurements and dark-field evaporation tests.

DSC method

The samples were placed in aluminum crucibles and measured in the temperature range from 25 °C to 110 °C, with a linear heating rate of 5 K min^{-1} and nitrogen flow flux of 20 mL min^{-1}. Table S1 shows the detailed parameters of the samples. As shown in Figure 4e, the DSC results show that the evaporation enthalpy of pure water and water in PPy-A membrane were 2452.38 kJ kg^{-1} and 1967.17 kJ kg^{-1} respectively, suggesting a reduction of vaporization enthalpy of water in PPy-A membrane. It should be noted that the DSC result present a fully dehydration process of the samples, and therefore the results might deviate from the enthalpy in actual evaporation process [4].

Table S1. Summary of DSC measurements.

	Pure water	Water in PPy-A
Total mass (mg)	4.48	4.31
Water mass (mg)	4.48	4.11
Enthalpy (kJ kg^{-1})	2452.38	1967.17

Dark-field evaporation

We further measured the evaporation enthalpy by dark-field evaporation. As shown in Figure S4a, the samples were placed in a sealed chamber with a stable temperature of 25°C in dark environment. The PPy membranes were hung on a PS foam to inhibit the downward evaporation. As shown in Figure S4b, the evaporation rate of water in PPy-A membrane was different from that of pure water, suggesting different evaporation mechanisms between the samples. We rationally assumed that the energy inputs for evaporation of pure water and water in PPy-A membrane were the same, which could be described by Equation (S2):

$$Q_{in} = h_{water} \cdot m_{water} = h_{PPy-A} \cdot m_{PPy-A} \quad \text{(S2)}$$

where Q_{in} is input energy, h_{water} is evaporation enthalpy of water, m_{water} is evaporation rate of pure water, h_{PPy-A} is evaporation enthalpy of water in PPy-A membrane, m_{PPy-A} is evaporation rate of water in PPy-A membrane. The equivalent evaporation enthalpy could be calculated based on the evaporation rate of water in different samples. The results in Figure S4b show that the equivalent evaporation enthalpy of PPy-A was calculated to be 1913.29 kJ kg^{-1}, which is slightly smaller than the DSC method owing the slightly dehydration of evaporation process. In addition,

PPy-F membrane exhibited an equivalent evaporation enthalpy of 171.29 kJ kg^{-1}, which is even smaller than the PPy-A membrane. The difference may be attributed to the vesicle-structure in another side of PPy-F membrane (as shown in Figure S3) and the doping of surfactants during the preparation of PPy-F membrane, which further weakens the hydrogen bond of water in PPy-F membrane. The specific mechanism still needs to be investigated in our future work. However, despite the PPy-F membrane exhibited reduced evaporation enthalpy, its evaporation rate was significantly lower than that of PPy-A membrane, further confirming the effectiveness of surface morphology manipulation in enhancing the evaporation performance.

Supporting Figure and Table

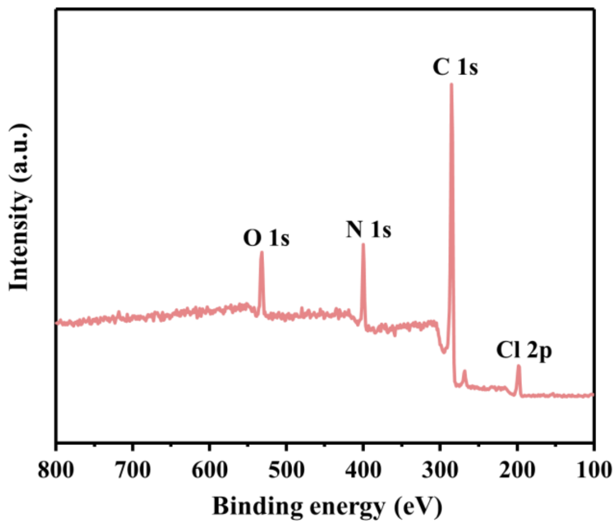

Figure S1. XPS spectrum of PPy-A membrane.

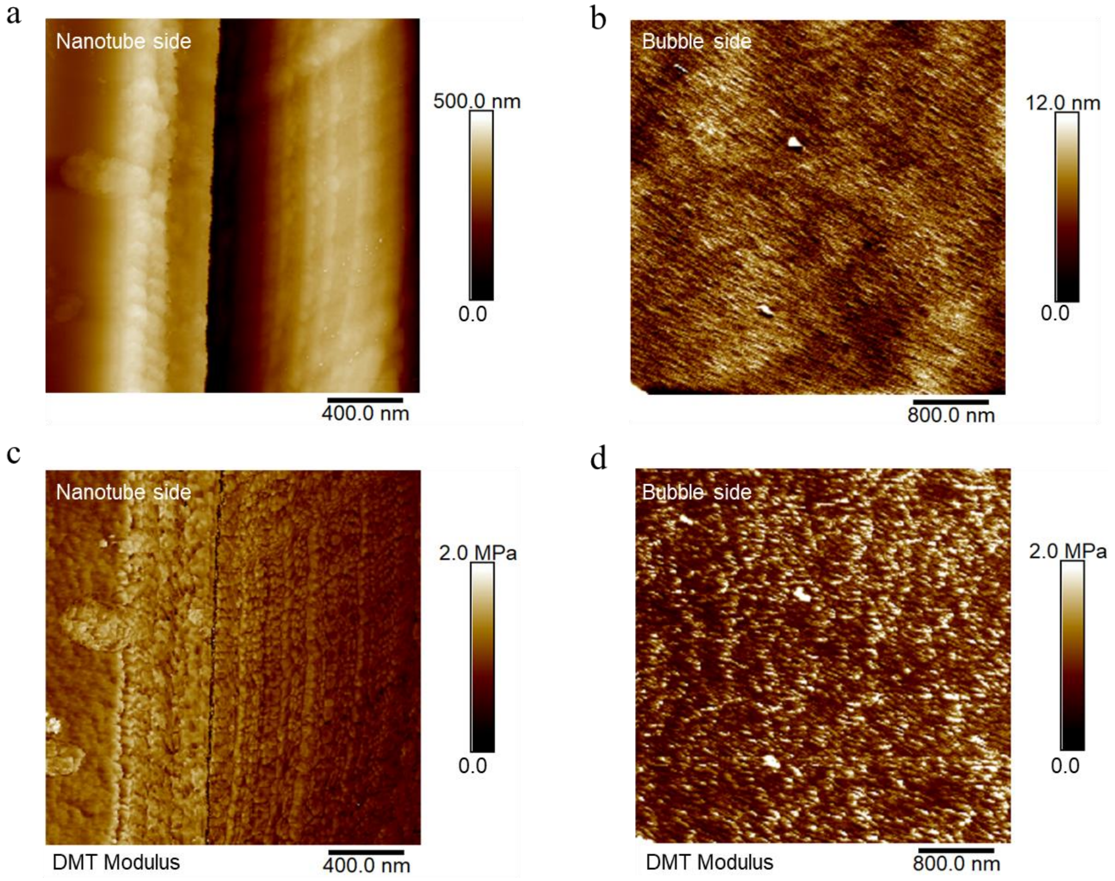

Figure S2. AFM images of (a) nanotube side and (b) bubble side of PPy-A membrane. DMT modulus images of (c) nanotube side and (d) bubble side of PPy-A membrane obtained by AFM.

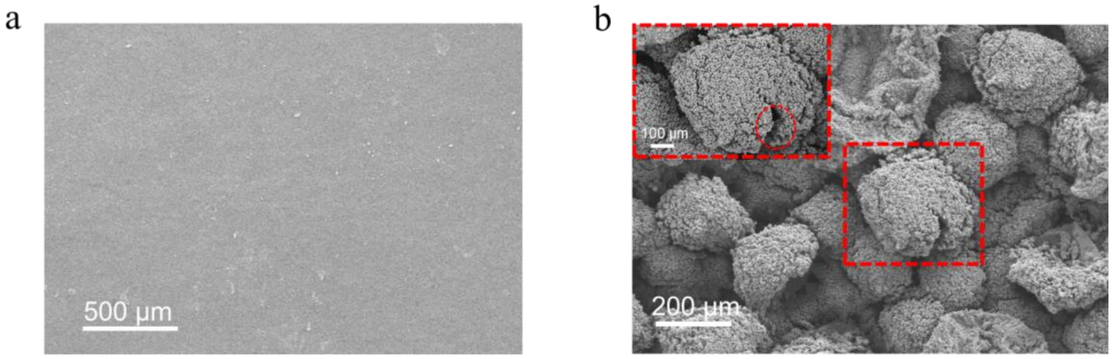

Figure S3. SEM images of (a) flat side and (b) vesicle side; inset is high-magnification image of the vesicle of PPy-F membrane.

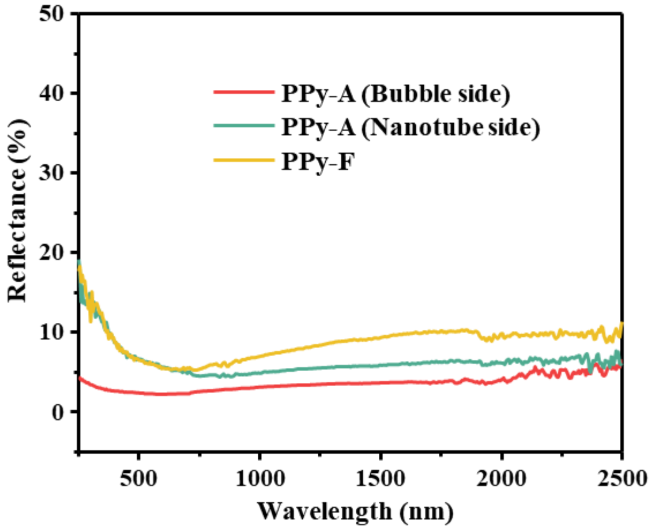

Figure S4. Reflection spectra of of bubble side and nanotube side of PPy-A membrane, and PPy-F membrane.

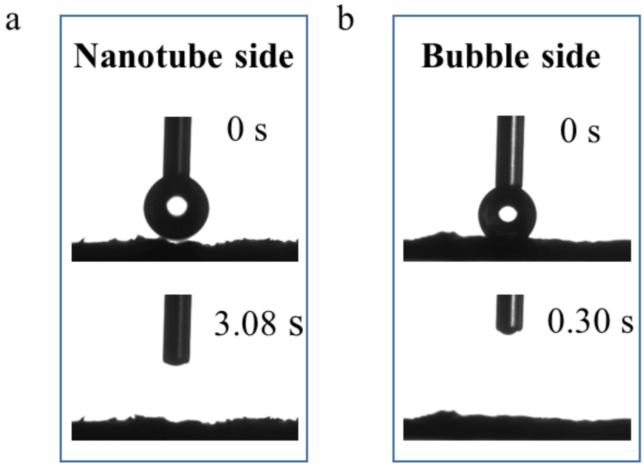

Figure S5. Dynamic water contact angle of (a) nanotube side and (b) bubble side of PPy-A membrane.

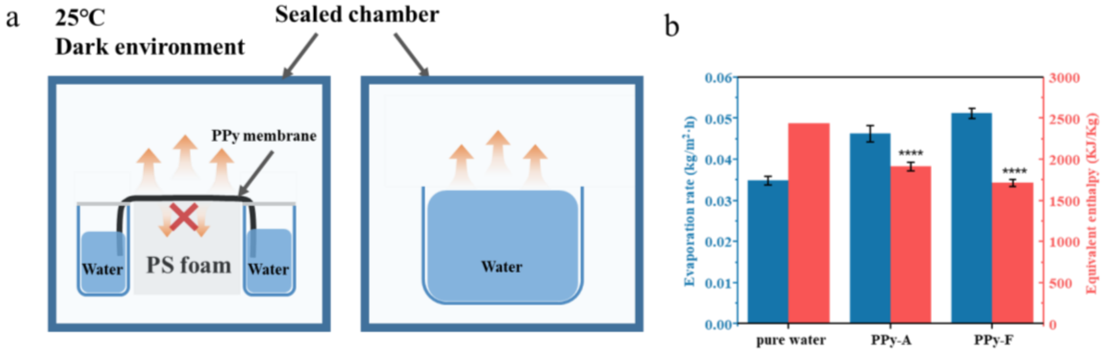

Figure S6. (a) Schematic illustration of dark-field test for pure water and PPy membrane. (b) Evaporation rate and equivalent enthalpy of pure water, PPy-A, and PPy-F membrane according to the dark-field evaporation test (pure water was set as control, ****p<0.0001, n=3).

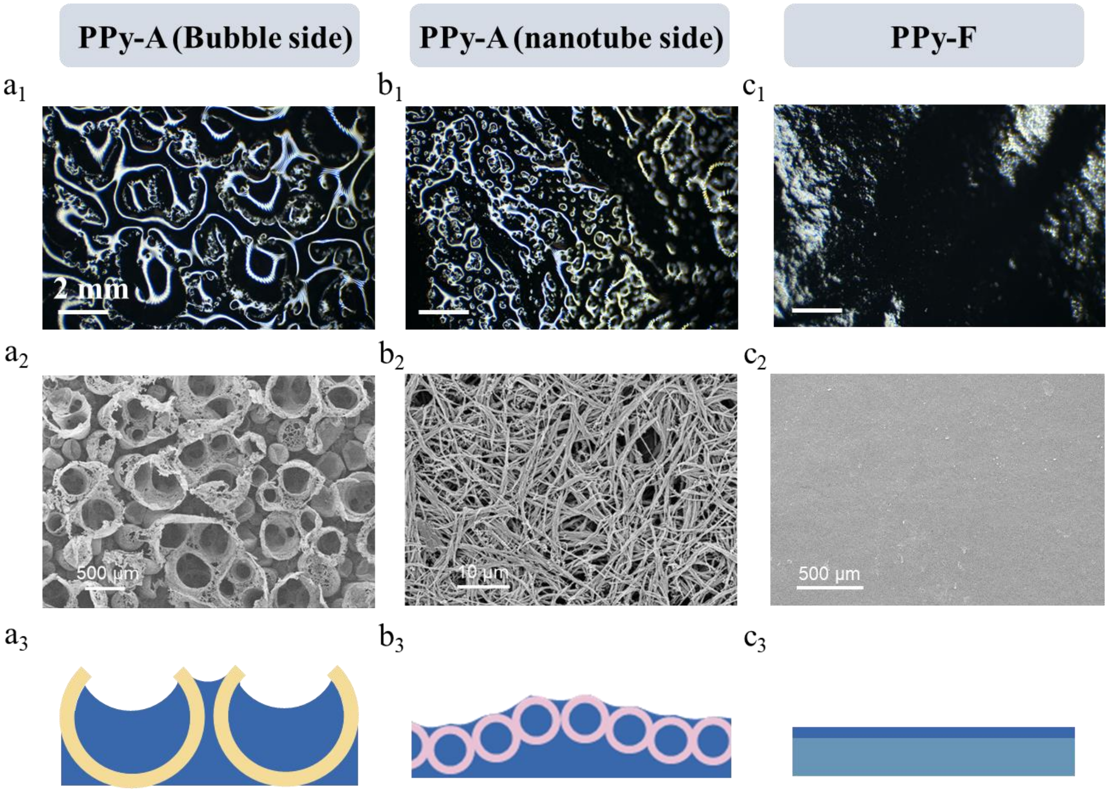

Figure S7. Aggregation state of water recorded with microscopy on the surface of (a_1) bubble side, (b_1) nanotube side of PPy-A membrane, and (c_1) PPy-F membrane during the natural evaporation process. SEM images of (a_2) bubble side, (b_2) nanotube side of PPy-A membrane, and (c_2) PPy-F membrane. Schematic illustration of water in (a_3) bubble side, (b_3) nanotube side of PPy-A membrane, and (c_3) PPy-F membrane.

Table S2. Natural evaporation rate of pure water, PPy-A membrane, and PPy-F membrane.

Evaporator	Pure water	PPy-A	PPy-F
Evaporation rate (kg m^{-2} h^{-1})	0.117	0.272	0.282

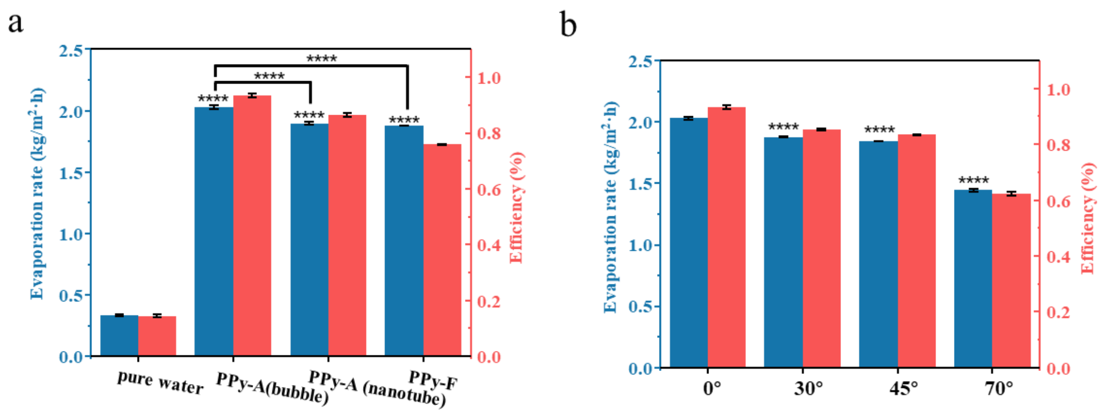

Figure S8. (a) Evaporation rate and energy efficiency of pure water, PPy-A membrane with bubble side and nanotube side under irradiation, and PPy-F membrane under 1 sun (pure water was set as control, ****p<0.0001, n=3)). (b) Evaporation rate and energy efficiency of PPy-A membrane with different tilt angles (0° was set as control, ****p<0.0001, n=3).

Table S3. Membrane thickness of PPy-A membrane with different polymerization time.

Polymerization time (h)	24	36	50	96
Membrane thickness (mm)	0.09	0.18	0.24	0.38

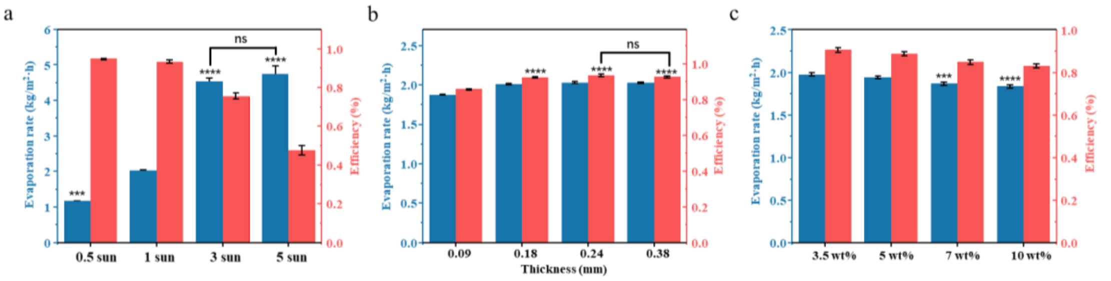

Figure S9. (a) Evaporation rate and energy efficiency of PPy-A membranes under 0.5, 1, 3, and 5 sun (1 sun was set as control, ****p*=0.001, *****p*<0.0001, *n*=3). (b) Evaporation rate and energy efficiency of PPy-A membranes with various membrane thickness (0.09 was set as control, *****p*<0.0001, *n*=3). (c) Evaporation rate and energy efficiency of PPy-A membranes in different brine salinity (3.5 wt% was set as control, ****p*=0.0006, *****p*<0.0001, *n*=3).

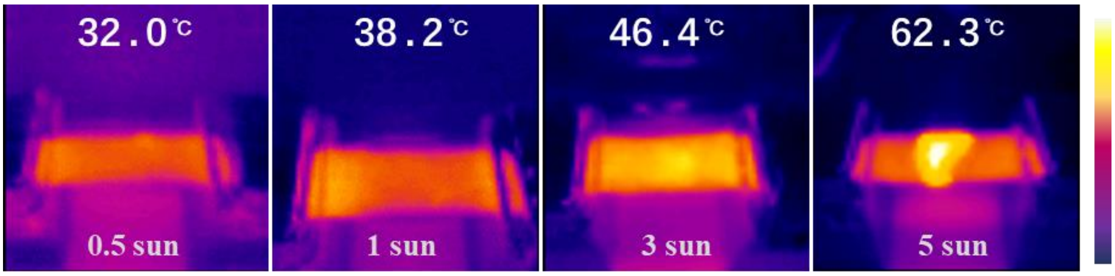

Figure S10. IR images of the PPy-A membrane in steady-state solar evaporation under 0.5 sun, 1 sun, 3 sun, and 5 sun irradiation.

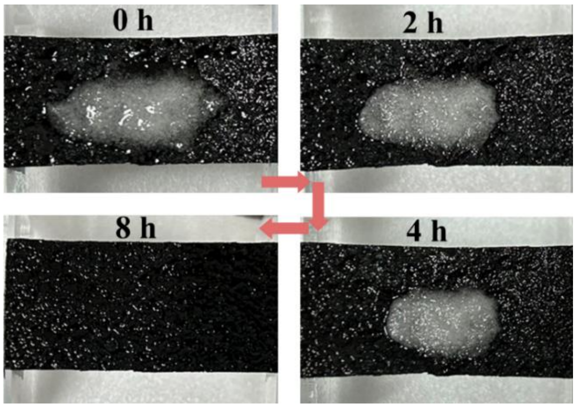

Figure S11. Dissolution of 0.5 g NaCl on the surface of PPy-A membrane with double-side water supply in dark.

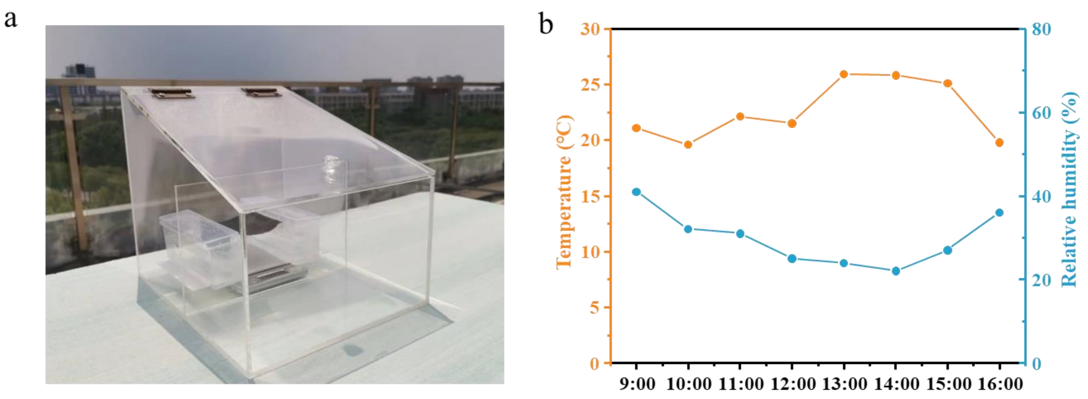

Figure S12. (a) Photograph of the outdoor desalination device. (b) Time-dependent environment temperature and humidity during the outdoor desalination.

Supporting References

[1] J. Mao, C. Li, H.J. Park, M. Rouabhia, Z. Zhang, Conductive Polymer Waving in Liquid Nitrogen, ACS Nano, 11 (2017) 10409-10416.10.1021/acsnano.7b05546

[2] Y. Li, L. Lan, F. Zhou, J. Peng, L. Guo, F. Wang, Z. Zhang, L. Wang, J. Mao, Flexible and easy-handling pristine polypyrrole membranes with bayberry-like vesicle structure for enhanced Cr(VI) removal from aqueous solution, J. Hazard. Mater., 439 (2022) 129598.https://doi.org/10.1016/j.jhazmat.2022.129598

[3] C.O. Popiel, J. Wojtkowiak, Simple Formulas for Thermophysical Properties of Liquid Water for Heat Transfer Calculations (from 0°C to 150°C), Heat Transfer Engineering, 19 (1998) 87-101.10.1080/01457639808939929

[4] F. Zhao, X. Zhou, Y. Shi, X. Qian, M. Alexander, X. Zhao, S. Mendez, R. Yang, L. Qu, G. Yu, Highly efficient solar vapour generation via hierarchically nanostructured gels, Nature Nanotechnology, 13 (2018) 489-495.10.1038/s41565-018-0097-z
